# Items

**Published:** 1897-05

**Authors:** 


### Items.

The unveiling of the Gross statue will take place at Washington, D. C., at 5 o'clock P. M., Wednesday, May 5, 1897. It will be remembered that some six years ago the president of the American Surgical Association, Dr. Claudius H. Mastin, of Mobile, in his annual address, proposed that a statue be erected to the memory of Prof. Samuel D. Gross, M. D., of Philadelphia. The culmination of this proposal will take place on the date above referred to, under the auspices of the American Surgical Association and the Alumni

Association of Jefferson Medical College, that have jointly issued invitations to the unveiling of the statue.

---

A MEDICAL press bureau has been established by Mr. Charles Wood Fassett in connection with the National Medical Association meetings. It will be held at Philadelphia in the medical exhibit hall, June 1–4, 1897. The membership fee is $5 and should be forwarded to Mr. Fassett, St. Joseph, Mo., or it can be paid to him at the meeting.

---

A SPECIAL tour to Europe and the International Medical Congress at Moscow, has been arranged under the patronage of Drs. N. Senn, Casey A. Wood, Harold N. Moyer, Eugene S. Talbot, D. R. Brower, J. B. Murphy, D. A. K. Steele and B. T. Whitmore, of Chicago. It is suggested that the comfort of those who intend visiting Moscow during the congress, August 19–26, 1897, will be greatly enhanced and the journey itself rendered more interesting and pleasant as well as more economical, by proceeding as one party instead of traveling singly or in small groups. Those wishing to travel in this way should communicate with Messrs. Cook & Son, 261 Broadway, New York, or 234 Clark street, Chicago. These well-known tourists' agents have arranged three itineraries, securing considerable reductions in the ordinary steamship, railway and hotel rates. An educated conductor will accompany each section of the party and the journey may be broken at almost any point to meet emergencies.

---

THE A. L. Hummel advertising agency, of New York, established by Dr. A. L. Hummel, of Philadelphia, whose province is exclusively medical journal advertising, has removed from 108 Fulton street to the Woodbridge building, 100 William street, N. Y.

---

THE New York Physicians' Mutual Aid Association makes a splendid showing in its twenty-eighth annual report, just issued. During the past year the families of eighteen physicians have been paid $1,000 each. There are 1,347 members, a net gain of fourteen during the year. The amount on hand January 1, 1897, was $32,627.69, distributed as follows: assessment fund, $3,307.79; contingent fund, $316.09; permanent fund, $29,003.81. The economic management of the association under Dr. Daniel D. Lewis, president, and his many capable coadjutors in the subordinate offices, deserves unstinted praise. The membership from Buffalo should be doubled during the present year.

